# SLUG is activated by nuclear factor kappa B and confers human alveolar epithelial A549 cells resistance to tumor necrosis factor-alpha-induced apoptosis

**DOI:** 10.1186/1477-7819-11-12

**Published:** 2013-01-22

**Authors:** Yaopeng Wang, Bin Yue, Xuyi Yu, Zhan Wang, Mingzhao Wang

**Affiliations:** 1 Department of Thoracic Surgery, the Affiliated Hospital of Medical College Qingdao University, 19 Jiangsu Road, Qingdao, Shandong, 266001, China; 2 Department of Surgery, the Affiliated Hospital of Medical College Qingdao University, 19 Jiangsu Road, Qingdao, Shandong, 266001, China; 3 Department of Medicine, Center Hospital of Qingdao City, 147 Siliu South Road, Qingdao, Shandong, 266001, China

**Keywords:** TNF-α, NF-κB, PUMA, SLUG, Bcl-2, apoptosis

## Abstract

**Background:**

The role of tumor necrosis factor alpha (TNF-α) in cancer is complex with both apoptotic and anti-apoptotic roles proposed. However the mechanism is not clear. In the study, we designed to investigate the effect of TNF-α on the activation and expression of nuclear factor kappa B (NF-κB)/p65/SLUG/PUMA/Bcl-2 levels in human lung cancer A549 cell line, and in conditions of TNF-α-induced apoptosis.

**Methods:**

We have engineered three A549 cell lines that were transiently transfected with PUMA siRNA, SLUG siRNA and Bcl-2 siRNA, respectively. We have measured the *in vitro* effects of siRNA on apoptosis, and sensitivity to 20 ng/ml of TNF-α treatment for 24–48 h.

**Results:**

We found the NF-κB activity and PUMA mRNA/protein was significantly increased after treatment of TNF-α for 24 h in untreated A549 cells, and led to a significant increase in TNF-α-induced apoptosis, no significant increase of SLUG and Bcl-2 level was shown. However, after treatment of TNF-α for 48 h in untreated A549 cells, SLUG and Bcl-2 level was significant increased, and PUMA level was significant decreased, and TNF-α-induced apoptosis was significantly decreased compared to the apoptosis level after treatment of TNF-α for 24 h. Inhibition of the NF-κB activity could effectively decrease the PUMA level and increase the SLUG and Bcl-2 level. PUMA silencing by siRNA led to a significant decrease in TNF-α-induced apoptosis after treatment of TNF-α for 24 h. Bcl-2 and SLUG silencing by siRNA led to a significant increase in TNF-α-induced apoptosis for 48 h. Furthermore, SLUG silencing increased PUMA level and decreased Bcl-2 level.

**Conclusions:**

The findings suggested that TNF-α treatment promoted apoptosis via the NF-κB-dependent PUMA pathway. The anti-apoptotic role of TNF-α was via NF-κB-dependent SLUG and Bcl-2 pathway at a later time.

## Background

TNF-α[GenBank:AAN76506] is a potent pleiotropic cytokine and is a major mediator of inflammation with multiple biological functions [1]. Several cancer therapies exploiting the cytotoxic effect of TNF-α on solid tumors and soft-tissue sarcomas have recently been examined in clinical trials [2,3]. TNF-α stimulates inflammation by turning on gene transcription through signaling cascades, such as the nuclear factor-kappa B (NF-κB)[GenBank:AAA20684] pathway [4]. This signaling also serves as the primary mechanism to protect cells against apoptotic stimuli [5,6]. In contrast, transcription factor NF-κB can also promote apoptosis in response to TNF-α through the activation of p53 upregulated modulator of apoptosis (PUMA)[GenBank:AAO16862] [7]. From these observations, it is possible to say that TNF-α has two different signaling pathways that contradict each other. The cytotoxic effect of TNF-α might be determined by ratios between the apoptosis-inducing and the apoptosis-inhibiting effects.

PUMA is a downstream target of p53 and a BH3-only B-cell CLL/lymphoma-2 (Bcl-2)[NCBI Reference Sequence:NR073605] family member. It is induced by p53 following exposure to DNA-damaging agents, such as γ-irradiation and commonly used chemotherapeutic drugs [7-9]. It is also activated by a variety of nongenotoxic stimuli independent of p53, such as serum starvation, kinase inhibitors, glucocorticoids, endoplasmic reticulum stress, and ischemia/reperfusion [10,11]. The pro-apoptotic function of PUMA is mediated by its interactions with anti-apoptotic Bcl-2 family members [12], which lead to mitochondrial dysfunction and caspase activation [13].

It has been reported that TNF-α-induced apoptosis is through the NF-κB-dependent PUMA pathway in colon cancer cells [7]. Simultaneous induction of the anti-apoptotic NF-κB targets by TNF-α, such as Bcl-2 and Bcl-extra large (Bcl-XL) was the cause of inhibition of the pro-apoptotic effect of PUMA [7].

However, in breast cancer, TNF-α resistance was also associated with SLUG[GenBank:GJ062364] upregulation [14]. In lung cancer, SLUG confers resistance to the epidermal growth factor receptor tyrosine kinase inhibitor [15]. SLUG, a snail family transcription factor, is also a suppressor of PUMA, which has been shown to be involved in the control of apoptosis and resistance of various cells to irradiation and chemotherapies [16-21]. However, knockdown of SLUG could effectively sensitize the cells to the stimulars above through PUMA upregulation [18-22]. Except for PUMA, SLUG downregulation facilitates apoptosis induced by pro-apoptotic drugs in neuroblastoma cells by downregulation of Bcl-2 prototypic anti-apoptotic protein [22]. We therefore suggested that SLUG plays an important role in the regulation of Bcl-2 and PUMA, by which to regulate the apoptotic effect. Storci *et al*. have shown that TNF-α upregulates SLUG via the NF-κB/HIF1alpha axis, which imparts breast cancer cells with a stem cell-like phenotype [23]. We therefore suggest that a significant relationship exists between NF-κB activation and SLUG/PUMA/Bcl-2.

Lung cancer is the most frequently occurring cancer in the world and causes more deaths in China. Despite advances in treatment modalities including radiation, surgery and chemotherapy, the overall survival in lung cancer remains low. TNF-α has been shown to regulate both apoptotic and anti-apoptotic pathways. In the present study, we investigated the effect of TNF-α on apoptosis in lung cancer A549 cells, and explored its mechanism. We demonstrated that TNF-α treatment promoted apoptosis via the NF-κB-dependent PUMA pathway at an early time. The anti-apoptotic role of TNF-α was via the Bcl-2 upregulation and PUMA downregulation by NF-κB-dependent SLUG upregulation at a later time.

## Methods

### Cell culture and antibodies

The human non-small cell lung cancer (NSCLC) cell line A549 was obtained from the American Type Culture Collection, and cultured in DMEM supplemented with 10% fetal bovine serum (FBS) and 100 IU/ml penicillin/streptomycin in a 37°C humidified incubator with 5% CO_2_. The inhibitor of nuclear factor kappa-B (IKK), wedelolactone, was obtained from Alexis Biochemical (Alexis Biochemical, Paris, France). Recombinant human TNF-α was from R&D Systems (Minneapolis, MN, USA). Antibodies for p65/PUMA/SLUG/Bcl-2/goat anti-rabbit IgG-HRP/goat anti-mouse IgG-HRP are from Santa Cruz Biotechnology (Santa Cruz, CA, USA). Anti-β-actin antibody were purchased from Sigma (Sigma-Aldrich, Shanghai, China), and Lamin A antibody from Abcam (Cambridge, MA, USA).

### siRNA transfection

Transfection was performed with Lipofectamine 2000 (Invitrogen, California, USA) following the manufacturer’s instructions. siRNA transfection was performed 24 h before 20 ng of TNF-α treatment. siRNA duplexes, including PUMA siRNA, SLUG siRNA and Bcl-2 siRNA and the control scrambled siRNA were from Santa Cruz Biotechnology.

### TNF-α treatment

Cells were plated in 12-well plates at 20 to 30% density 24 h before treatment. Human TNF-α (20 ng) was diluted with appropriate cell culture media. In some experiments, cells were pretreated with 25 μM of the IKK inhibitor, wedelolactone, for 12 h before addition of 20 ng of TNF-α.

### RT-PCR analysis

Total RNA was extracted from cultured cells with Isogen (Nippon Gene, Tokyo, Japan) according to the manufacturer’s instructions. Random-primer, first strand cDNA was generated from 0.5 μg total RNA with a Takara RNA PCR kit (Takara, Japan), and the PCR analysis was performed using gene-specific primers and first strand cDNA as the template. The sequence of primers was as follows: SLUG, 5^′^-gacacacatacagtgattatt-3^′^ and 5^′^-aaacttttcagcttcaatggc-3^′^; PUMA, 5^′^-cgacctcaacgcacagtacga-3^′^, and 5^′^-aggcacctaattgggctccat-3^′^; Bcl-2, 5^′^-cgacgacttctcccgccgctaccgc-3^′^; reverse, 5^′^-ccgcatgctggggccgtacagttcc-3^′^; GAPDH, 5^′^-acatcgctcagacaccatgg-3^′^ and 5^′^-gtagttgaggtcaatgaaggg-3^′^. The PCR products were fractionated on a 2% agarose gel and visualized after ethidium bromide staining. After 3 minutes at a temperature of 95°C, the experimental reaction consisting of 30 cycles at 95°C for 30 s, 58°C for 60 s, and 72°C for 30 s, and the PCR products were analyzed by gel electrophoresis. The relative expression of mRNA was quantitatively measured using densitometry analysis by gene expression analysis software.

### Western blot analysis

Cytoplasmic and nuclear fractions from cells were prepared using the NE-PER nuclear and cytoplasmic Extraction kit (Thermo scientific Pierce, Rockford, IL, USA). Cells were lysed with RIPA buffer (150 mM NaCl, 1.0% Nonidet P-40, 0.5% sodium deoxycholate, 0.1% SDS, 50 mM Tris, pH 8.0) containing protease inhibitor cocktail (Roche Applied Science, Mannheim, Germany) and 2 mM sodium vanadate. Protein concentration was determined by BCA protein assay reagent kit (Pierce). Equal amounts of cell lysates were separated by 10% SDS-PAGE, electrophoretically transferred to nitrocellulose membrane (Pall, Pensacola, FL, USA), immunoblotted with primary antibody against p-p65/P65/SLUG/PUMA/Bcl-2/β-actin. Blots were visualized with anti-rabbit or anti-mouse IgG conjugated with horseradish peroxidase (HRP) and ECL reagents (Pierce).

### Electrophoretic mobility shift assay

Cell extracts were prepared using a commercially available nuclear extraction kit according to the manufacturer’s protocol (Pierce). Electrophoretic mobility shift assay was performed according to the provided protocol (Promega, Madison, WI, USA). In brief, a 21-mer oligonucleotide corresponding to the consensus NF-κB site (Promega) was radiolabeled with (γ-32P)-ATP by aT4 kinase reaction. Nucleotides were purified by chromatography through a G-25 spin column (Roche Diagnostics Co. West Sussex, UK) equilibrated in TE buffer. Ten micrograms of nuclear protein extract were incubated with the radiolabeled NF-κB oligonucleotide for 20 minutes at room temperature. For competition studies, a 50-fold molar excess of unlabeled oligonucleotide was added to the reaction (a 21-mer corresponding to the AP-1 consensus sequence (Promega) was used to confirm specificity). DNA protein complexes were separated by electrophoresis through a nondenaturing 4% polyacrylamide gel in 0.5 × TBE at 100 V for 2.5 h. Autoradiographic films were developed after 18 h of exposure to the gels (−20°C).

### Fluorescence-activated cell sorting (FACS) analysis

To identify the induction of apoptosis, treated cells underwent propidium iodide (PI) staining and FACS as to the manufacture’s instruction. In brief, cells were plated at a density of 1 × 10^5^ cells/ml. After allowing 24 h for cell adherence, cells were transfected and/or treated. Cells were collected by gentle trypsinization, washed in PBS, pelleted by centrifugation and fixed in 70% ethanol. Immediately prior to staining, cells were washed twice in PBS and resuspended in PBS containing RNase (20 μg/ml). Cells were stained with PI (final concentration 10 μg/ml) for 10 minutes at room temperature. Samples were analyzed by FACS (FL-3 channel) using a Beckman Coulter Counter Epics XL flow cytometer (Beckman Coulter, Miami, FL, USA). For each sample, 50,000 events were collected and stored for subsequent analysis using EXPO software (version 2.0; Applied Cytometry Systems, Sheffield, UK). The percentage of cells in the sub-G0 phase was quantitated as an estimate of cells undergoing apoptosis.

### Statistical analysis

All statistical analyses were performed using the SPSS10.0 software. The results were presented as means ± SD of three replicate assays. Differences between groups were assessed using analysis of variance (ANOVA) or the Dunnett *t*-test. A *P*-value < 0.05 was considered to indicate statistical significance.

## Results

### TNF-α treatment of A549 cells upregulates SLUG with a dependency on NF-κB activation

**Figure 1:**
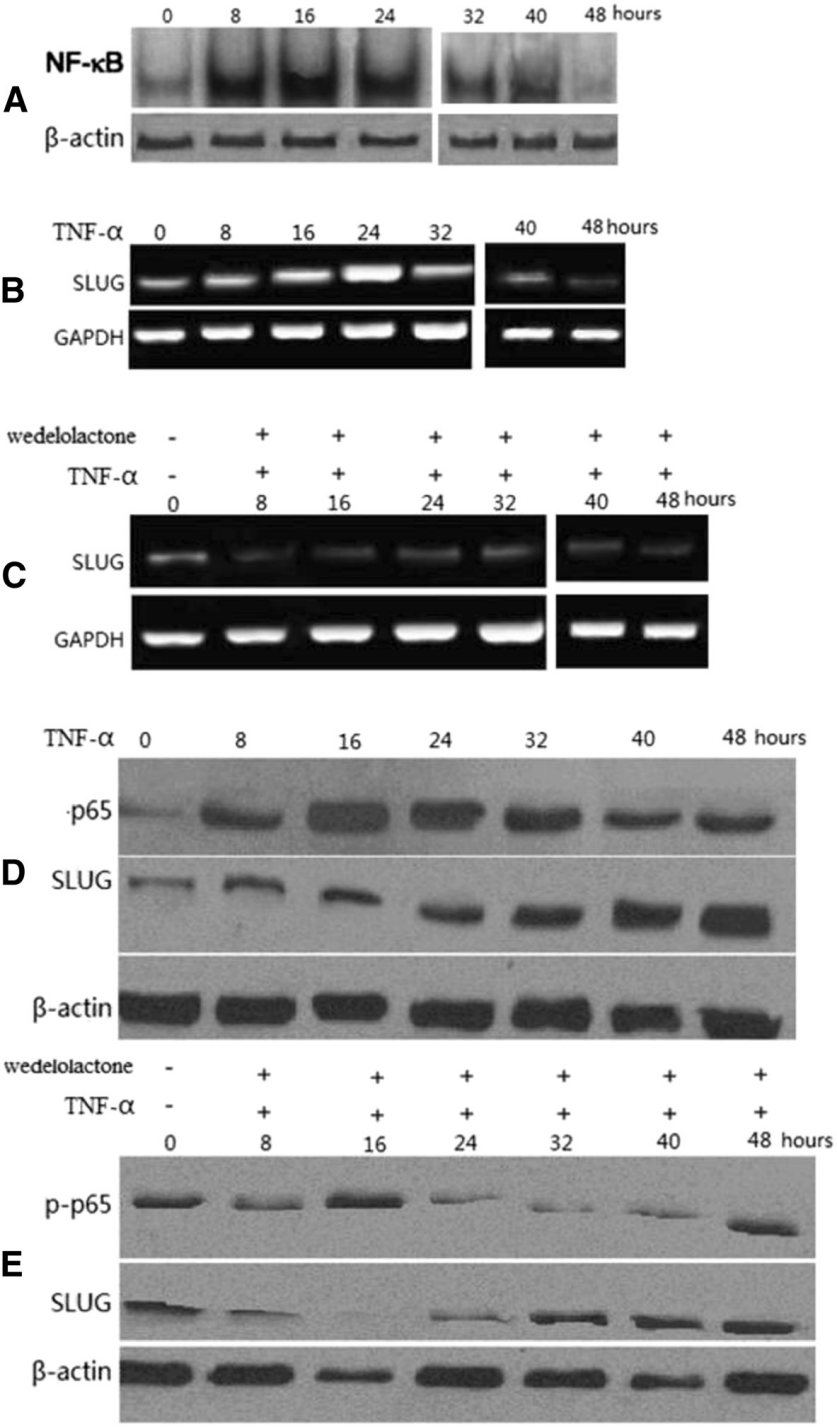
**Effect of TNF-α on nuclear factor kappa B (NF-κB) and SLUG expression.** Lung cancer cells were treated with 20 ng/ml TNF-α. NF-κB transactivation function was determined by electrophoretic mobility shift assay. SLUG mRNA and protein at different time points after treatment were analyzed by reverse transcription PCR(RT-PCR) and western blotting, respectively. (**A**) NF-κB transactivation function was determined by electrophoretic mobility shift assay at different time points after TNF-α treatment. (**B**) Time course of SLUG mRNA induction by TNF-α in A549 lung cancer cells. Glyceraldehyde-3-phosphate dehydrogenase (GAPDH) was a control. (**C**) Time course of SLUG mRNA induction with or without TNF-α/wedelolactone in A549 lung cancer cells. GAPDH was a control. (**D**) Time course of SLUG protein induction by TNF-α in A549 cells. Phospho-p65 and β-actin were also analyzed. (**E**) Time course of SLUG and phospho-p65 protein induction with or without TNF-α/wedelolactone in A549 lung cancer cells.

Treatment with 20 ng/ml of TNF-α significantly stimulated NF-κB transactivation function as determined by the electrophoretic mobility shift assay (Figure 1A). The induction of NF-κB activity was increased 8 h after treatment, with the peak level of NF-κB activity at 16 h. The stimulation of NF-κB appeared to decrease after 16 h of treatment with TNF-α. To determine the role of SLUG in TNF-α-induced apoptosis, lung cancer cells were treated with 20 ng/ml of TNF-α. Both SLUG mRNA and protein were induced by TNF-α within several hours, with the peak level of SLUG mRNA induction at 24 h (Figure 1B), and that of protein at 48 h (Figure 1C). To determine whether NF-κB activation is involved in SLUG induction, lung cancer cells were pretreated with the pharmacological IKK inhibitor, wedelolactone, then treated with TNF-α. Induction of SLUG mRNA and protein was significantly inhibited (Figure 1D and E). The result indicated TNF-α treatment resulted in SLUG induction in lung cancer cell lines via NF-κB activation.

### TNF-α treatment of A549 cells upregulates Bcl-2 is dependent on NF-κB-dependent-SLUG

**Figure 2:**
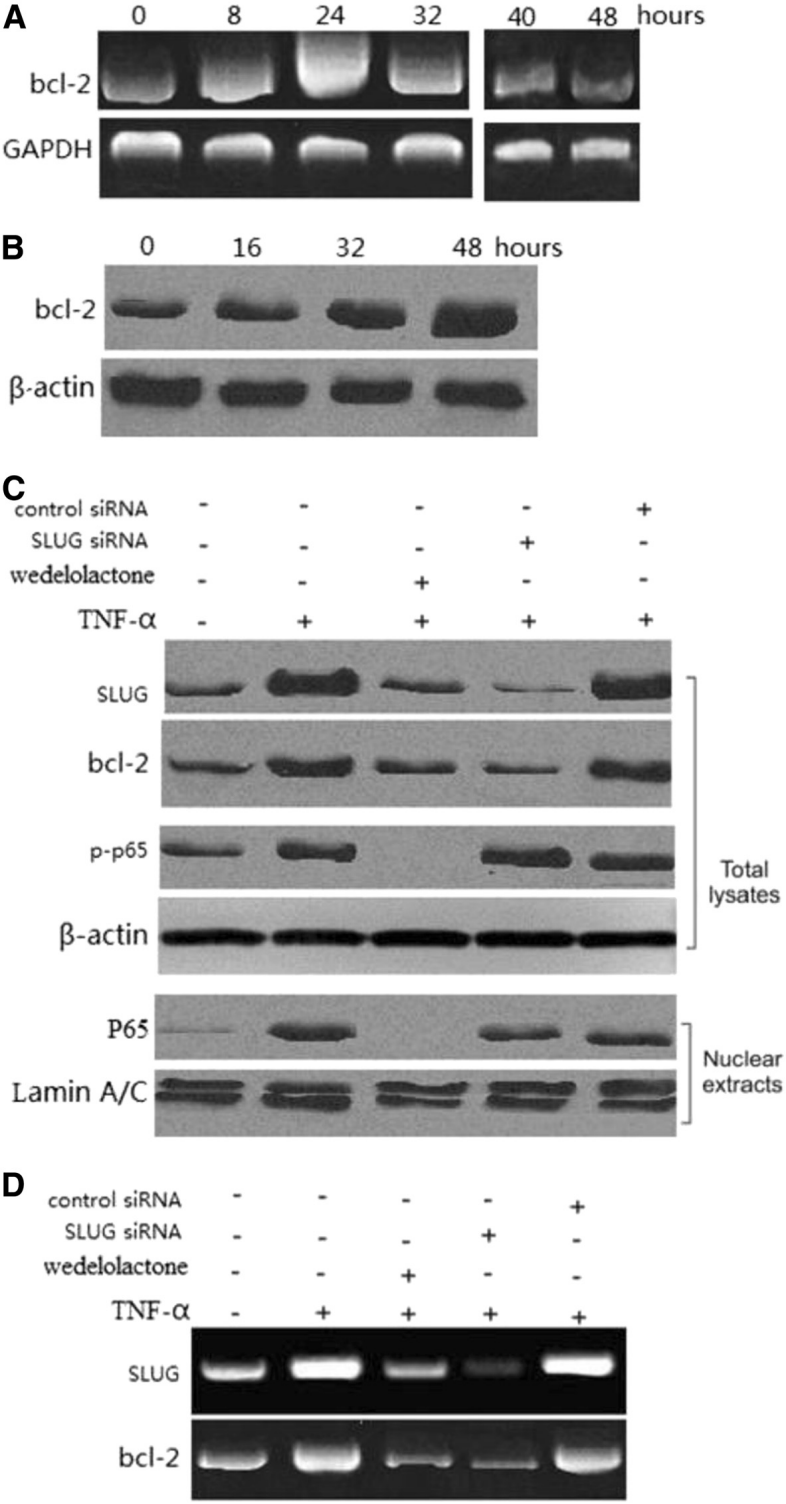
**Effect of TNF-α on Bcl-2 expression.** Lung cancer cells were treated with 20 ng/ml TNF-α. (**A**) Time course of Bcl-2 mRNA induction by TNF-α in A549 lung cancer cells. Glyceraldehyde-3-phosphate dehydrogenase (GAPDH) was loaded as control. (**B**) Western blot analysis of Bcl-2 protein expression in A549 cells followed TNF-α treatment for different times. (**C**) Wedelolactone-pretreated A549 lung cancer cells were transfected with SLUG or the control scrambled siRNA, and then treated with TNF-α for 48 h. SLUG, Bcl-2 and p-P65 expression was analyzed by western blot. (**D**) Wedelolactone-pretreated A549 lung cancer cells were transfected with SLUG or the control scrambled siRNA, and then treated with TNF-α for 24 h. SLUG, Bcl-2 and p-P65 expression was analyzed by reverse transcription PCR.

Treatment with 20 ng/ml of TNF-α significantly induced Bcl-2 mRNA at 24 h (Figure 2A), and Bcl-2 protein at 48 h (Figure 2B), as with SLUG. To determine whether NF-κB-dependent SLUG upregulation is necessary for the induction of Bcl-2 by TNF-α, we used pharmacological IKK inhibitor, wedelolactone, which is responsible for NF-κB activation. Wedelolactone pretreatment abrogated IκBα phosphorylation and degradation following TNF-α treatment, and suppressed subsequent SLUG and Bcl-2 induction and p65 nuclear translocation (Figure 2C and D). Furthermore, pretreatment with SLUG siRNA following TNF-α treatment significantly inhibited Bcl-2 levels (Figure 2C and D). The above data collectively indicate that SLUG-dependent induction of Bcl-2 by TNF-α is mediated by NF-κB activation.

### TNF-α treatment of A549 cells upregulation of PUMA is dependent on NF-κB activation

**Figure 3:**
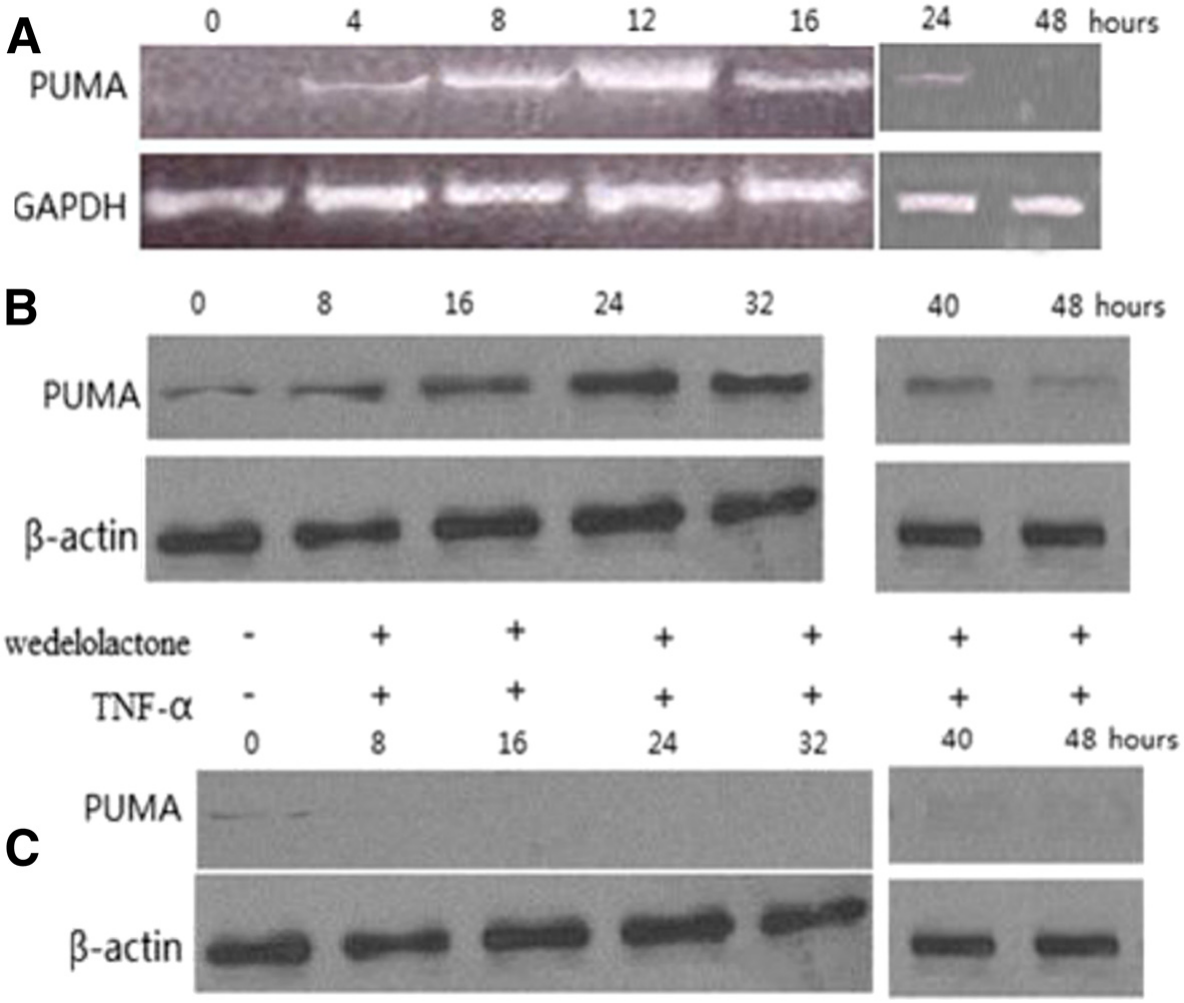
**Effect of TNF-α on p53 upregulated modulator of apoptosis (PUMA) expression.** (**A**) Reverse transciption PCR analysis of PUMA mRNA expression in A549 cells following TNF-α treatment. (**B**) Time course of PUMA protein induction by TNF-α in A549 cells. (**C**) Induction of PUMA protein by TNF-α or wedelolactone treatment for different times.

A549 cells were treated with 20 ng/ml of TNF-α. Both PUMA mRNA and protein were induced by TNF-α within several hours, with the peak level of PUMA mRNA induction at 12 h (Figure 3A), and that of protein at 24 h (Figure 3B). A significant decrease in PUMA protein was found at 48 h with 20 ng/ml of TNF-α treatment (data not show). To determine whether NF-κB is necessary for the induction of PUMA by TNF-α, we used wedelolactone to inhibit NF-κB activation. Following downregulation of NF-κB activity, PUMA was blocked with TNF-α treatment. The above data indicate that induction of PUMA by TNF-α is mediated by p65 through the canonical NF-κB pathway.

### TNF-α treatment of A549 cells induces apoptosis with a dependency on PUMA activation

**Figure 4:**
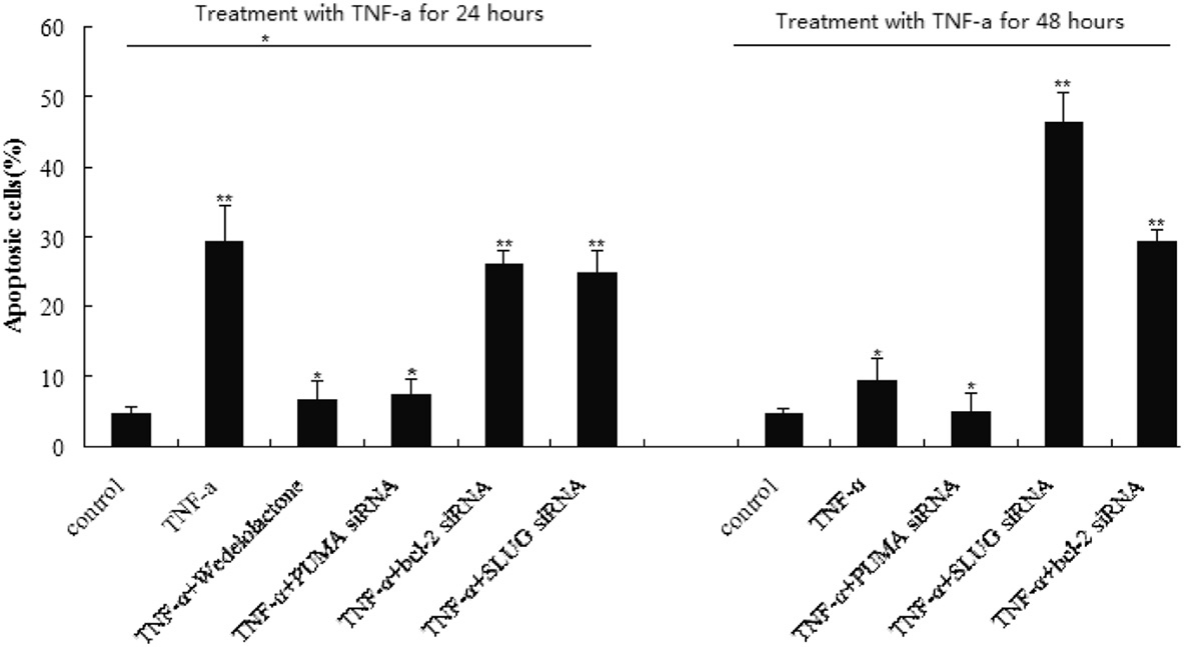
**Effect of p53 upregulated modulator of apoptosis (PUMA), Bcl-2 and SLUG on apoptosis of TNF-α-induced apoptosis.** Apoptpsis of A549 cells following TNF-α treatment were analyzed after treated with wedelolactone, PUMA, bcl-2 or SLUG siRNA.

Thirty percent of apoptosis was detected in lung cancer cells following 20 ng/ml of TNF-α treatment for 24 h (Figure 4). However, for 48 h treatment, the apoptosis rate reduced to 10% (Figure 4). Wedelolactone pretreatment or knockdown of PUMA by siRNA led to a significant decrease in TNF-α-induced apoptosis for 24 h (Figure 4, ^*^P > 0.05, ^**^P < 0.01). The results suggest that NF-κB-mediated PUMA induction represents a novel mechanism mediating TNF-α-induced apoptosis.

### SLUG silencing of A549 cells increases TNF-α-induced apoptosis via PUMA activation

**Figure 5:**
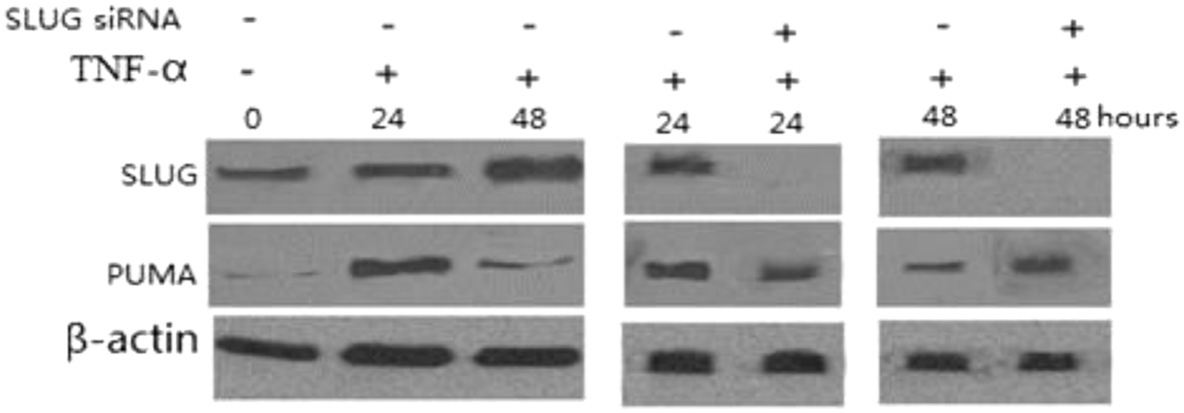
PUMA expression was analyzed by SLUG siRNA treatment following TNF-α treatment.

Thirty percent of apoptosis was detected in lung cancer cells following 20 ng/ml of TNF-α treatment for 24 h. However, only a low level (< 10%) of apoptosis was detected in lung cancer cells following TNF-α treatment at 48 h. This might be due to simultaneous induction of the anti-apoptotic NF-κB targets by TNF-α, such as SLUG and Bcl-2 at 48 h (Figure 1 and 2), in addition to PUMA. In Figure 1 both SLUG mRNA and protein were induced by TNF-α within several hours, with the peak level of SLUG mRNA induction at 24 h (Figure 1), and that of protein at 48 h (Figure 1). Indeed, knockdown of SLUG by siRNA led to a significant increase in TNF-α-induced apoptosis (Figure 4, *P > 0.05, **P < 0.01). Our study demonstrated that SLUG silencing promoted PUMA expression induced by TNF-α (Figure 5). The results suggest that a low level (< 20%) of apoptosis at 48 h was in part due to SLUG upregulation, which suppressed the PUMA upregulation of TNF-α induced apoptosis.

### SLUG silencing of A549 cells increases TNF-α-induced apoptosis via Bcl-2 inactivation

In addition to PUMA, anti-apoptotic Bcl-2 was also upregulated with TNF-α treatment. Knockdown of Bcl-2 by siRNA also led to a significant increase in TNF-α-induced apoptosis, consistent with the previous finding that Bcl-2 is the major survival factor in lung cancer cells. These results suggest that overexpression of Bcl-2 can compromise TNF-α-induced and PUMA-mediated apoptosis (Figure 4, *P > 0.05, **P < 0.01).

## Discussion

The role of TNF-α in cancer is complex with both apoptotic and anti-apoptotic roles proposed. In this study, we have shown TNF-α promoted apoptosis *in vitro* in A549 cells for 24 h, and inhibited apoptosis in A549 cells for 48 h.

Following TNF-α treatment of 20 ng/ml for 24 h, we have detected 30% of apoptosis in lung cancer A549 cells, during which, NF-κB transactivation function was significantly stimulated. Simultaneous induction of the pro-apoptotic PUMA mRNA/protein was also found, and the induction of PUMA by TNF-α is mediated by p65 through the canonical NF-κB pathway. Otherwise, wedelolactone pretreatment to inhibit NF-κB activity or knockdown of PUMA by siRNA led to a significant decrease in TNF-α-induced apoptosis for 24 h, which was consequent to Wang’s report [7]. Our study suggested TNF-α treatment promotes apoptosis through the NF-κB-dependent PUMA pathway at an early time in lung cancer A549 cells. Activation of NF-κB is known to render cancer cells resistant to anticancer drugs. Inhibition of NF-κB has been explored as an attractive approach for anticancer therapies [24]. However, our data suggest that NF-κB inhibition can compromise PUMA induction by inflammatory cytokines, which may be involved in tumor suppression and be beneficial for anticancer therapies.

TNF-α is not an effective inducer of apoptosis in cultured cells, likely due to simultaneous induction of both pro-apoptotic and anti-apoptotic proteins by NF-κB. In the present study, TNF-α is an effective inducer of apoptosis in cultured A549 cells for 24 h, a phenomenon which may be cell-specific. However, only low levels (<10%) of apoptosis were detected in A549 cells following TNF-α treatment for 48 h. We found the PUMA mRNA/protein was significantly decreased after TNF-α treatment for 48 h, and the anti-apoptotic proteins Bcl-2 and SLUG, which have been shown to be involved in the control of apoptosis and resistance of various cells to irradiation and chemotherapies was significantly increased [16-21].

SLUG, a member of the snail superfamily of zinc finger transcription factors, is the key epithelial-mesenchymal transition (EMT) regulator responsible for conferring acquired resistance to target therapy in lung cancer [25]. Previous studies showed that the expression of SLUG promotes the invasivity of lung cancer cells through increased activity of metalloproteinase-2 and suppression of E-cadherin [26]. A recent report suggested transfection of c-Kit in parental multiple myeloma (MM) cells in the presence of stem cell factor (SCF) up-regulated SLUG and increased resistance to the chemotherapeutic agents. Moreover, MM cells expressing SLUG showed a similar increased resistance to the chemotherapeutic agents [27]. Vitali *et al*. had suggested that reducing the expression of SLUG enhanced the sensitivity of neuroblastoma cell lines to imatinib mesylat by attenuating Bcl-2 expression [22]. SLUG can also antagonize p53-mediated apoptosis in hematopoietic progenitors by repressing PUMA [17]. It has been reported that knockdown of SLUG sensitizes cancer cells to irradiation and Cisplatin by PUMA upregulation [18,19]. Furthermore, knockdown of SLUG could effectively sensitize the cells to the stimulars above through PUMA upregulation [18-22]. Thus, SLUG could regulate cancer cell survival via direct or indirect transcriptional regulation of pro-apoptotic and anti-apoptotic genes, although further study will be required to resolve the molecular details.

Our study showed TNF-α treatment for 48 h promoted SLUG and Bcl-2 levels and decreased PUMA levels followed by decreased apoptosis in A549 cells. After siRNA knockdown of SLUG or Bcl-2, apoptosis of TNF-α-induced for 48 h was significantly increased. Otherwise, knockdown of SLUG significantly upregulated the PUMA level and decreased the Bcl-2 level. We therefore suggest that TNF-α treatment for 48 h increased the NF-κB-dependent SLUG upregulation, by which PUMA was inhibited and Bcl-2 level promoted, and then inhibited the apoptosis in A549 cells.

## Conclusions

We demonstrated that PUMA is a target of NF-κB and a critical mediator of TNF-α-induced apoptosis. At an early time, TNF-α treatment induced apoptosis by PUMA activity. At a later time, TNF-α-induced apoptosis was inhibited by NF-κB-dependent SLUG upregulation, which suppressed PUMA and increased Bcl-2 activity.

## Abbreviations

ANOVA: analysis of variance; Bcl-2: B-cell CLL/lymphoma-2; Bcl-XL: Bcl-extra large; DMEM: Dulbecco’s modified Eagles medium; EMT: epithelial-mesenchymal transition; FBS: fetal bovine serum; GAPDH: glyceraldehyde-3-phosphate dehydrogenase; HRP: horseradish peroxidase; IKK: inhibitor of nuclear factor kappa-B; MM: multiple myeloma); NF-κB: nuclear factor-kappa B; NSCLC: non-small cell lung cancer; PBS: phosphate-buffered saline; PCR: polymerase chain reaction; PI: propidium iodide; PUMA: p53 upregulated modulator of apoptosis; SCF: stem cell factor; TNF-α: tumor necrosis factor alpha.

## Competing interests

The authors declare that they have no competing interests.

## Authors’ contributions

YW carried out the studies, carried out the experiment of molecular biology and drafted the manuscript. BY participated in the experiment. XY participated in the sequence alignment. ZW participated in the design of the result and performed the statistical analysis. MW coordinated and helped to draft the manuscript. All authors read and approved the final manuscript.
